# Plasma Membrane Ca^2+^-ATPase Isoforms Composition Regulates Cellular pH Homeostasis in Differentiating PC12 Cells in a Manner Dependent on Cytosolic Ca^2+^ Elevations

**DOI:** 10.1371/journal.pone.0102352

**Published:** 2014-07-11

**Authors:** Tomasz Boczek, Malwina Lisek, Bozena Ferenc, Antoni Kowalski, Dariusz Stepinski, Magdalena Wiktorska, Ludmila Zylinska

**Affiliations:** 1 Department of Molecular Neurochemistry, Medical University, Lodz, Poland; 2 Department of Molecular Biology and Genetics, Aarhus University, Aarhus, Denmark; 3 Department of Cytophysiology, University of Lodz, Lodz, Poland; 4 Department of Molecular Cell Mechanisms, Medical University, Lodz, Poland; Instituto Nacional de Cardiologia I. Ch., Mexico

## Abstract

Plasma membrane Ca^2+^-ATPase (PMCA) by extruding Ca^2+^ outside the cell, actively participates in the regulation of intracellular Ca^2+^ concentration. Acting as Ca^2+^/H^+^ counter-transporter, PMCA transports large quantities of protons which may affect organellar pH homeostasis. PMCA exists in four isoforms (PMCA1-4) but only PMCA2 and PMCA3, due to their unique localization and features, perform more specialized function. Using differentiated PC12 cells we assessed the role of PMCA2 and PMCA3 in the regulation of intracellular pH in steady-state conditions and during Ca^2+^ overload evoked by 59 mM KCl. We observed that manipulation in PMCA expression elevated pH_mito_ and pH_cyto_ but only in PMCA2-downregulated cells higher mitochondrial pH gradient (ΔpH) was found in steady-state conditions. Our data also demonstrated that PMCA2 or PMCA3 knock-down delayed Ca^2+^ clearance and partially attenuated cellular acidification during KCl-stimulated Ca^2+^ influx. Because SERCA and NCX modulated cellular pH response in neglectable manner, and all conditions used to inhibit PMCA prevented KCl-induced pH drop, we considered PMCA2 and PMCA3 as mainly responsible for transport of protons to intracellular milieu. In steady-state conditions, higher TMRE uptake in PMCA2-knockdown line was driven by plasma membrane potential (Ψp). Nonetheless, mitochondrial membrane potential (Ψm) in this line was dissipated during Ca^2+^ overload. Cyclosporin and bongkrekic acid prevented Ψ_m_ loss suggesting the involvement of Ca^2+^-driven opening of mitochondrial permeability transition pore as putative underlying mechanism. The findings presented here demonstrate a crucial role of PMCA2 and PMCA3 in regulation of cellular pH and indicate PMCA membrane composition important for preservation of electrochemical gradient.

## Introduction

Neuronal differentiation is associated with spatially and temporary coordinated elevations in cytosolic Ca^2+^ concentration - (Ca^2+^)_c_ - propagated due to Ca^2+^ entry via plasma membrane and its release from internal stores [1], [2]. These physiological and pathological Ca^2+^ signals are modulated by the activity of mitochondria, which buffer (Ca^2+^)_c_ and regulate Ca^2+^-dependent activation or inhibition of several processes [3], [4]. For example, mitochondrial control of Ca^2+^ signal is crucial for regulation of both the cell membrane's voltage and, especially, for pH gradients driving ATP generation [5]. Mitochondria not only link Ca^2+^ homeostasis to cell metabolism, but may also drive cell fate by controlling ATP/ADP ratio.

Acting as the energetic centers, they shape signaling pathways, control propagation of Ca^2+^ waves and by providing ATP to calcium pumps boost calcium gradients [6]. Elevations of Ca^2+^ in the mitochondrial matrix regulate voltage (ΔΨ_m_, negative inside) and pH (ΔpH, alkaline inside) components of electrochemical gradient. According to the chemiosmotic model, ΔΨ_m_ and ΔpH are thermodynamically equivalent to power ATP synthesis [7]. Even though ΔpH constitutes only 20–30% of proton motive force, it is essential for electroneutral transport of ions and movement of metabolites into the matrix [8]. The electrical gradient establishes most of the potential difference. Together with ΔpH, it sets the driving force for ATP synthase, and for cytosolic Ca^2+^ to enter the matrix [9]. Moderate elevations of Ca^2+^ in the matrix activate dehydrogenases of Krebs cycle, modulate the activity of electron transport chain and stimulate the respiratory rate [6], [10]. This may make mitochondrial membrane more negative. On the other hand, Ca^2+^ overload may activate permeability transition pore (mPTP) formation allowing ions to leave the mitochondrion, thereby triggering cell death [9].

Mitochondrial Ca^2+^ uptake in intact cells was observed at low cytosolic Ca^2+^ concentrations ranging from 150 to 300 nM [11]. However, elevations in (Ca^2+^)_c_ stimulate matrix acidification and result in ΔpH drop what is suggested to decrease oxygen consumption [12]. The newest finding located plasma membrane calcium pump (PMCA) in the center for intracellular protons transport [13]. Because PMCA operates as Ca^2+^/H^+^ counter-transport with a 1∶1 stoichiometry, the extrusion of Ca^2+^ generates large quantities of protons that are transmitted to mitochondrial matrix leading to pH decrease [13]. Since Ca^2+^ and protons have opposite effects on many cellular processes, the role of PMCA in the regulation of calcium homeostasis may be of fundamental importance for preservation of cellular energy.

PMCA exists in four isoforms PMCA1-4. Pumps 1 and 4 are ubiquitously distributed and perform a “housekeeping” role whereas the location of 2 and 3 isoforms is restricted to only some tissues where they perform more specialized functions [14]–[16]. Due to the abundance of PMCA2 and PMCA3 in the nervous system they are termed neuron-specific. During development their expression undergoes considerable changes reflecting the importance of the spatial organization of Ca^2+^ extrusion systems for synaptic formation [17]–[19]. Moreover, the observation of mRNA distribution suggests that the expression of PMCA2 and PMCA3 is controlled by different mechanisms than the two other isoforms [20]. The studies on PMCA have made clear that unique PMCA2 properties distinguish it from other basic isoforms. It possesses the highest resting activity and calmodulin sensitivity, and represents more than 30–40% of the total pump protein in mature neurons [21]. Thus, PMCA2 is thought to be the principal ATPase that maintains Ca^2+^ homeostasis following neural excitation. The existence of PMCA2 is expected to provide neuronal cells with higher sensitivity to even subtle (Ca^2+^)_c_ changes. This specificity of PMCA2, which is further highlighted by its interaction with specific partners [22], could explain why this isoform plays a predominant role in neuronal cells that have special Ca^2+^ demands. The role of PMCA3 is much less understood. However, distribution, kinetic properties and scarce studies including our previous work on PC12 cells suggest that it should be also considered as an important Ca^2+^ player in differentiation process.

To study the potential role of neuro-specific PMCA isoforms in regulation of cellular pH, we used differentiated PC12 lines with experimentally downregulated PMCA2 or PMCA3. Due to possessing of several features characteristic for sympathetic-like neurons [23], this cell line is an excellent model system to study neuronal processes. We found that PMCA2- or PMCA3-deficient cells maintained higher pH_mito_ and pH_cyto_ but only in PMCA2-downregulated line increased ΔpH was observed in steady-state conditions. Also, we demonstrated that PMCA2 and PMCA3 were primarily responsible for Ca^2+^-dependent pH_mito_ and pH_cyto_ decreases and accompanying ΔpH drop during KCl stimulations. In PMCA2-downregulated cells, Ca^2+^ overload led to dissipation of mitochondrial membrane potential, a phenomenon that was blocked by cyclosporin and bongkrekic acid suggesting the involvement of mitochondrial permeability transition pore. Our findings point out that neuro-specific PMCA isoforms are important regulators of cellular pH in steady-state conditions and may also shape Ca^2+^-dependent pH changes during depolarization events.

## Materials and Methods

### Reagents

All reagents, if not separately mentioned, were purchased from Sigma-Aldrich (Germany). The PC12 rat pheochromocytoma cell line was obtained from ATCC (USA) and from Sigma-Aldrich (Germany). RPMI 1640 medium was from PAA (Austria). Calf and horse sera were from BioChrom (UK). Maxima SYBR Green Master Mix was from Fermentas (Canada). M-MLV Reverse Transcriptase, Trizol, Alexa Fluor 488, MitoTracker Red 580, MitoTracker Green TM, Fura-2 AM and SNARF were from Life Technologies (USA). Total RNA isolation kit was from Epicentre Biotech. (USA). Protein Assay Kit was from Bio-Rad (USA). TurboFect transfection regent was from Thermo Scientific. Primary antibodies against GFP, GAPDH, PMCA2 and PMCA3 were from Santa Cruz Biotech. (USA). Paq5000 polymerase was from Stratagene (USA). Phosphothioate oligodeoxynucleotides were from IDT (USA). Primers were synthesized in Institute of Biochemistry and Biophysics (Poland). mitoSypHer construct was kindly donated by Dr. Nicolas Demaurex.

### The model of stable transfection

pcDNA3.1(+) vectors carrying the antisense oligonucleotides directed to either PMCA2 or PMCA3 were used to establish a stably-transfected PC12 lines as described in [24]. Cells were cultured as described previously [25] and differentiated with 1 mM dibutyryl-cAMP for 48 h. All the results presented here were obtained following 2-day differentiation process. For pH measurements, mitoSypHer probe was transfected to undifferentiated antisense-carrying PC12 lines with TurboFect Transfection reagent and 2 days later cells were differentiated as described above. Routinely, the expression of PMCA2, PMCA3 and mitoSypHer was controlled by real-time PCR every two passages and no more than 6 passages were used. To increase the accuracy and maintain the reproducibility of the data we separately transfected two PC12 lines of different sources. The description of the lines as _2 (PMCA2-deficient line), _3 (PMCA3-deficient line) and C (mock transfected line) was adapted.

### Transient transfection

PC12 cells transient transfection with antisense probes described in [26] and listed in Table 1 was conducted using TurboFect transfection reagent. In brief, three phosphothioate oligodeoxynucleotides antisense to translated regions of mRNA of either PMCA2 or PMCA3 were added in equimolar concentrations (4 µM) to a serum-free RPMI medium. Total concentration of oligodeoxynucleotides was kept at 12 µM during transfection. After 6 h, medium was replaced with complete RPMI medium and cells were allowed to recover for another 48 h. After recovery period transfection was repeated. Cells transfected with a mismatch oligonucleotide sequence (12 µM) was used as a control for antisense oligonucleotides transfection. Growth medium and reagents were changed in all culture flasks at the same time. Following second transfection, the efficiency of PMCA knock-down was assessed by Western blot determination of PMCA protein level. Cells were differentiated as described in *The model of stable transfection* section.

**Table 1:** Antisense phosphothioate oligodeoxynucleotides used for PMCA2 or PMCA3 knock-down in a model of transient transfection.

Primer	Sequence
PMCA2-1	5′-C*CTTGGGCCGTGGCACATCCTTCATTGCT*C-3′
PMCA2-2	5′-G*GTGAGCTTGCCCTGAAGCACCGACTTCT*C-3′
PMCA2-3	5′-C*CAGCAGGCCACACTCTGTCTTGTTGCCC*A-3′
PMCA3-1	5′-C*TGCCCATAGATCTGCCTGCGTTTCTCCAAGT*C-3
PMCA3-2	5′-A*CCCGGCCATCCACAACGAAGGTCTCAATCAC*A-3′
PMCA3-3	5′-T*TACCATGTCATCCCGGTCCCGAGGACGAAAT*C-3′
Mismatch	5′- TGTGAATCTGTTAGCCTTAACCTTAAGTTC-3′

### Viability assay

Cellular viability was assessed using WST-1 assay. 5×10^3^ cells were seeded in a 96-well plate and incubated with WST-1 solution in a 1∶10 ratio for 4°C at 37°C. The absorbance of samples was spectrophotometrically measured at 450 nm.

### pH measurements

For mitoSypHer/SNARF dual imaging, cells expressing mitoSypHer were adhered to poly-L-lysine coated coverslips and differentiated for 2 days. Then, the culture medium was changed into a buffer containing 20 mM HEPES, 131 mM NaCl, 5 mM KCl, 1 mM MgCl_2_, 10 mM glucose, 2.2 mM CaCl_2_, 10 mM NaHCO_3_ and 1 mM KH_2_PO_4_ at pH 7.4 and cells were loaded with 10 µM SNARF (with 0.01% pluronic acid) for 40 min at 37°C. Simultaneous pH_mito_/pH_cyto_ measurements were performed in a thermostatic chamber at 37°C on TCS SP5 laser scanning confocal microscope equipped with DM6000 CFS system, DFC 360FX camera, HCX PL APO 63× objective and LAS AF Lite software (Leica). Fluorescence imaging was done with the tandem resonant scanner (16 kHz bidirectional, ∼25 frames/s). SNARF and mitoSypHer were excited using argon laser low-intensity light (488 nm). The fluorescence emitted in the range of 500–530 nm was collected for mitoSypHer whereas fluorescence in two separate channels (620–765 nm and 560–600 nm) was collected for SNARF. At the end of each experiment, fluorescence changes were calibrated to absolute mitochondrial and cytosolic pH using nigericin (5 µg/ml) and monensin (5 µM) in pH 9.5–10.0 (20 mM N-methyl-D-glutamine), pH 8.0–9.0 (Tris), pH 7.0–7.5 (HEPES) or pH 5.5–6.5 (MES), as described in [13]. The calibration curve was fitted to sigmoidal equation using GraphPad Prism 5.01. The emission ratio (620 nm–765 nm)/(560 nm–600 nm) for SNARF was calculated in MetaFluor 6.3 (Universal Imaging) and processed in MS Excel. The measured bleedthrough between SNARF and mitoSypHer probe was less than 4%, as evaluated using online Fluorescence SpectraViewer software. Unless otherwise indicated, inhibitors were added 20 min before measurement. The role of NCX (Na^+^/Ca^2+^ exchanger) in KCl-evoked pH changes was assessed in a loading buffer with 0–5 mM Na^+^. Ca^2+^- free solution contained 1 mM EDTA instead of 2.2 mM CaCl_2_.

### Single-cell Ca^2+^ imaging

Cells expressing mitoSypHer were adhered to poly-L-lysine coated slides and loaded with 10 µM Fura-2 for 1 h at 37°C. After several washes, cells were placed in a buffer composed as described in *pH measurements* section. When recording simultaneously with mitoSypHer, Fura-2 was alternately excited at 340 and 380 nm for 0.3 s through a 505 nm dichroic long pass filter and 535 nm emission filter. When recording only Fura-2, the dye was excited at 340 and 380 nm (0.3 s) through a 430 nm long way pass dichroic filter and a 510 nm bandpass emission filter. The mitoSypHer was alternately excited at 430 and 480 nm for 0.3 s through a 505 nm dichroic long pass filter and imaged with a 535 nm band pass emission filter. The contour of single cells was taken to define region of interest (ROI) from which the fluorescence was recorded. Background fluorescence was automatically subtracted from all measurements. Ratiometric images of pH_mito_ and Ca^2+^ were acquired using fluorescent Axiovert S100 TV inverted microscope (Carl Zeiss) equipped with a 40× Plan Neofluar objective and attached to a cooled CCD camera (Spectral Instruments Inc.).

### Immunocytochemistry

Confocal microscopy was used to analyze SypHer mitochondrial targeting and mitochondrial mass. ∼10^3^ differentiated cells seeded on poly-L-lysine coated glass LabTek II chamber slides were fixed for 30 min with 3.8% paraformaldehyde and permeabilized with 0.1% Triton X-100 for 10 min at 4°C. Fixed cells were then blocked with 6% bovine serum albumin (BSA), overnight incubated with monoclonal anti-GFP antibody (1∶100) at 4°C and probed with secondary antibodies conjugated to Alexa Fluor 488 (1∶1000) for 2 h at room temperature. Next, mitochondria were stained for 15 min with MitoTracker Red 580 (40 nM). Images were taken on TCS S5 confocal laser scanning microscope with 63× objective (Leica). In a separate experiment, mitochondrial mass was determined with MitoTracker Green. In this method cells were first loaded with 150 nM MitoTracker Green FM for 30 min at 37°C and then fixed as described above. The average fluorescence intensity after background subtraction was measured with TCS S5 microscope accompanying software (Leica). Raw images were processes with CorelDraw Graphics Suite 11.

### Total cell lysate preparation and Western blot analysis

Scraped cells were resuspended in RIPA buffer supplemented with 1 mM PMSF, 2 mM Na_3_VO_4_ and protease inhibitor cocktail and lysed for 30 min on ice. Then, lysates were centrifuged at 10 000× g for 5 min and supernatants were boiled for 5 min in Laemmli buffer. Total protein content was quantified using Bio-Rad Protein Assay.

20 µg of total cellular proteins were resolved on a 10% SDS-PAGE and transferred onto nitrocellulose membrane using semi-dry method. Membranes were first blocked with 6% BSA in TBS-T buffer (10 mM Tris-HCl, pH. 7.4, 150 mM NaCl, 0.05% Twwen-20) for 1 h at room temperature and then probed overnight at 4°C with primary antibodies against GFP (1∶1000), PMCA2 (1∶750), PMCA3 (1∶750) and GAPDH (1∶1000). Following several washes with TBS-T, membranes were incubated with appropriate secondary antibodies (1∶5000) coupled with alkaline phosphatase at room temperature for 4 h. Bands were visualized using Sigma Fast BCIP/NBT used according to the manufacturer's instructions. Blots were scanned and the bands intensity was measured using GelDocEQ with Quantity One 1-D Analysis Software version 4.4.1 (Bio-Rad).

### RNA isolation and PCR reactions

Total cellular RNA was extracted using Trizol reagent according to the procedure provided by the manufacturer. 1 µg of isolated RNA was subsequently used for cDNA synthesis with oligo(dT) primers in a 20 µl reaction mixture containing M-MLV reverse transcriptase. The cDNA templates were used to quantify gene expression level using Maxima SYBR Green Master Mix in the following conditions: 15 min at 95°C followed by 40 cycles at 95°C for 15 s, 60°C for 30 s and 72°C for 30 s. PCR reactions were performed in an AbiPrism 7000 sequence detection system (Applied Biosciences). For each PCR amplicon, a melting curve was run. The relative fold change after normalization to Gapdh expression was calculated using a comparative 2^−ΔΔCt^ method [27].

Conventional PCR used to estimate the efficiency of mitoSypHer transfection was carried out using Paq5000 polymerase in the following conditions: 5 min at 95°C followed by 30 cycles at 95°C for 1 min, 50°C for 1 min, 72°C for 2 min with a final extension at 72°C for 10 min in T300 thermocycler (Biometra) using cDNA obtained as described above. PCR products after staining with ethidium bromide were analyzed under UV light in GelDocEQ system (Bio-Rad). The primers used in PCR reactions are listed in Table 2.

**Table 2:** Primers used in PCR reactions.

Gene	Primer sequences
PMCA2	F:5′-ACCGTGGTGCAGGCCTATGT-3′;R:5′-GGCAATGGCGTTGACCAGCA-3′
PMCA3	F:5′-AGGCCTGGCAGACAACACCA-3′;R:5′-TCCCACACCAGCTGCAGGAA-3′
Tfam	F:5′-GAAAGCACAAATCAAGAGGAG-3′;R:5′-CTGCTTTTCATCATGAGACAG-3′
Nrf-1	F:5′-TTACTCTGCTGTGGCTGATGG-3′;R:5′-CCTCTGATGCTTGCGTCGTCT-3′
Pgc-1α	F:5′-GTGCAGCCAAGACTCTGTATGG-3′;R:5′-GTCCAGGTCATTCACATCAAGTTC-3′
CcO-I	F:5′-GGGCATCCATGCAGTCATTCTAG-3′;R:5′-GCGGGGATACCTCGTCGTT-3′
CcO-III	F:5′-ATGGTTTCGGTTACCTTCTATTA-3′;R:5′-CAGCCTAGTTCCTACCCACGAC-3′
Hyper	F:5′-GAGCAAAGACCCCAACGAGA-3′;R:5′-AGCGCTGGCAGTAAAGTGAT-3′
Gapdh	F:5′-GGTTACCAGGGCTGCCTTCT-3′;R:5′-CTTCCCATTCTCAGCCTTGACT-3′

Tfam – mitochondrial transcription factor A; Nrf-1 – nuclear respiratory factor 1; Pgc-1α – peroxisome proliferator-activated receptor-gamma coactivator 1 alpha; CcO-I – cytochrome c oxidase subunit I; CcO-III – cytochrome c oxidase subunit III.

### Determination of mitochondrial swelling

Mitochondria were isolated as described in [28]. The experiments were carried out at 30°C in a reaction medium containing 200 mM sucrose, 10 mM HEPES, pH 7.4, 10 µM EGTA, 5 mM KH_2_PO_4_, 2 µM rotenone (to inhibit electron backflow to complex I), 1 µg/ml oligomycin (to maintain constant ATP/ADP ratio) and mitochondria suspended at ∼1 mg/ml. Before exposure to 10 µM CaCl_2_, mitochondria were energized with 5 mM succinate for 2 min. Cyclosporin (1 µM), bongkrekic acid (10 µM) or atractylate (20 µM) was added just prior to succinate. Swelling was assessed by changes in light scattering monitored spectrophotometrically at 520 nm under a continuous stirring of mitochondrial suspension.

### Monitoring of mitochondrial and plasma membrane potential (ΔΨ_m_ and ΔΨ_p_)

Mitochondrial membrane potential (ΔΨ_m_) was measured with TMRE (tetra-methyl-rhodamine-ethyl ester), which accumulates in mitochondrial matrix according to the Nernst equation [29], whereas plasma membrane potential (ΔΨ_p_) was measured with DiSBAC_2_ (Bis-(1,3-diethylthiobarbituric acid)trimethine oxonol). For estimation, cells were loaded in a dark with 25 nM TMRE or 1 µM DiSBAC_2_ for 30 min at 37°C in a buffer A containing 20 mM HEPES, pH 7.4, 2 mM CaCl_2_, 150 mM NaCl, 5 mM KCl, 1 mM MgCl_2_, 10 mM glucose and analyzed by FACScan Becton Dickinson flow cytometer using an accompanying software. Cells incubated with 0.1% DMSO, used as a solvent for TMRE, were monitored to record background fluorescence, which was later subtracted from the TMRE recordings. Changes in ΔΨ_m_ were monitored in resting cells and at selected points following 59 mM KCl treatment (10 min after first KCl addition, recovery, 10 min after second KCl addition, recovery). The reliability of TMRE to be used for ΔΨ_m_ measurement was confirmed by the pre-incubation with either 6 µM oligomycin or 1 µM FCCP (carbonyl cyanide p-trifluoromethoxyphenylhydrazone) for 10 min. The influence of ΔΨ_p_ on mitochondrial TMRE uptake was assessed by 5 min preincubation of the cells with Ca^2+^-free buffer A containing 59 mM KCl before loading with 25 nM TMRE. Cyclosporin A (1 µM), bongkrekic acid (10 µM) or FK-506 (10 µM) were added to the culture medium 1 h before 25 nM TMRE loading.

TMRE fluorescence decay in single cells was assessed using TILL Photonics dual wavelength imaging system equipped with Polychrome IV monochromator (TILL Photonics GmbH). TMRE-loaded cells (25 nM) were illuminated at 535 nm through a 15 nm band-pass filter for 2 min and following 30 s depolarization with 1 µM FCCP. Fluorescence at 580 nm was recorded with equipped CCD camera (Spectral Instruments Inc.). Digital camera and monochromator were controlled by TILL Vision 4.0 imaging software, which was also used for data collection and processing. All procedures were performed at 37°C.

### Statistical analysis

The data are shown as means ± SEM of n separate experiments (n≥3). Statistical analyses were done using STATISTICA 8.0 (StatSoft). Normally distributed data were analyzed with one-way ANOVA with Tukey's post-hoc test. In other cases, Kruskal-Wallis non-parametric ANOVA with post-hoc Dunn's test was applied. *P*-value <0.05 was considered as statistically significant.

## Results

### The knockdown of PMCA2 or PMCA3 in stably transfected differentiated PC12 cells

**Figure 1:**
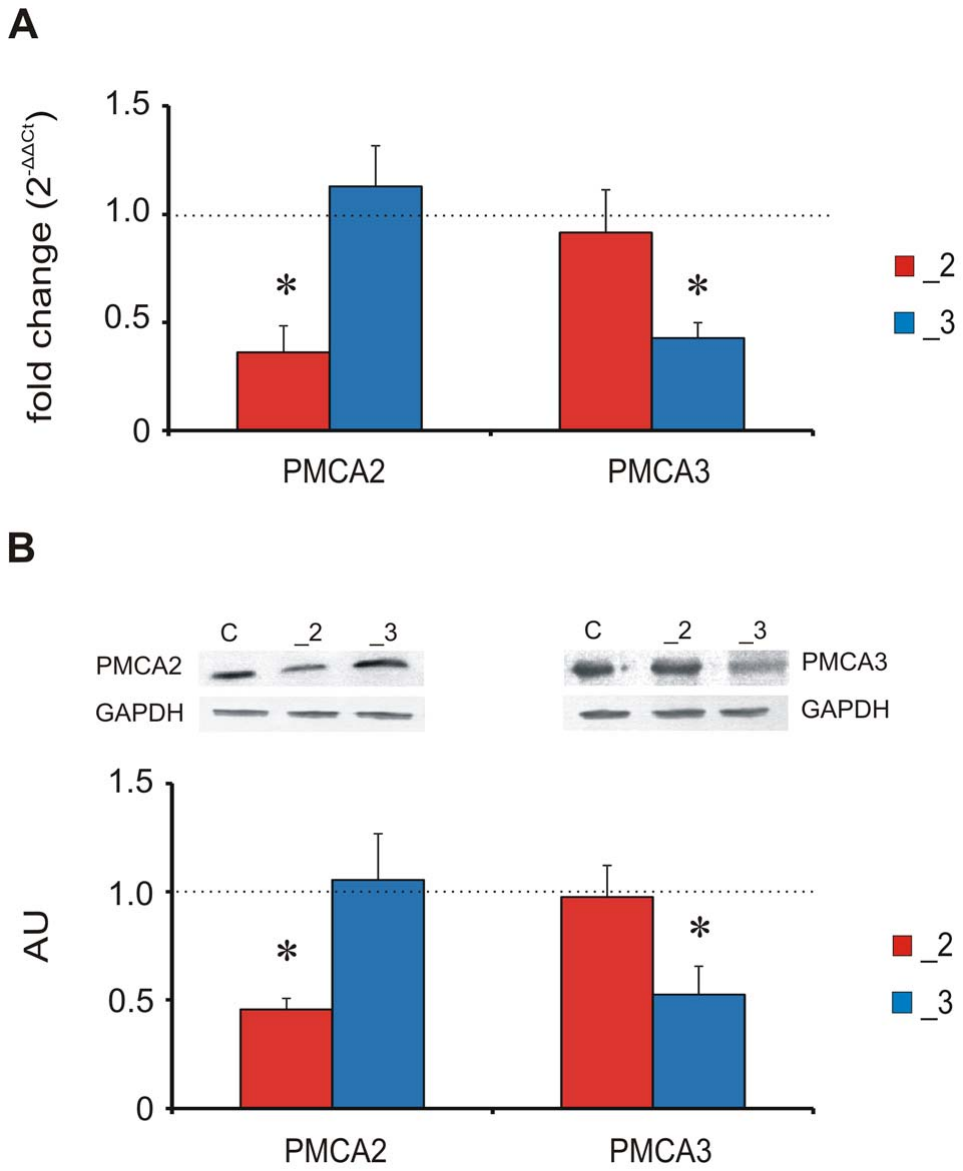
The efficiency of PMCA knockdown in a model of stable transfection. (A) The expression of genes corresponding to PMCA2 or PMCA3 was assessed using real-time PCR. The results are presented as relative units obtained after normalization to Gapdh expression. The level of expression of each target gene in control line was taken as 1 (dotted line). * P<0.05, PMCA-deficient lines vs. control cells. (B) PMCAs protein level assessed by densitometric analysis of immunoblots. The results are presented as arbitrary units (AU) obtained after normalization to endogenous GAPDH level. The dotted line presents the values for control line. * P<0.05, PMCA-deficient lines vs. control cells.

The expression of PMCA2 or PMCA3 mRNA was mostly abolished (∼60% decrease) by an antisense mRNA targeted against it, but was not changed in mock-transfected cells (Fig. 1A). Similarly, PMCA2 or PMCA3 protein level was not affected by mock transfection but was decreased by ∼50% following transfection with antisense-carrying vectors (Fig. 1B). Both PMCA2 or PMCA3 mRNA and proteins were normalized to endogenous GAPDH mRNA and protein levels, respectively. Unless otherwise stated, the experiments were performed using stably transfected lines.

### PMCA2- and PMCA3-deficient cells maintain higher cytosolic and mitochondrial pH

**Figure 2:**
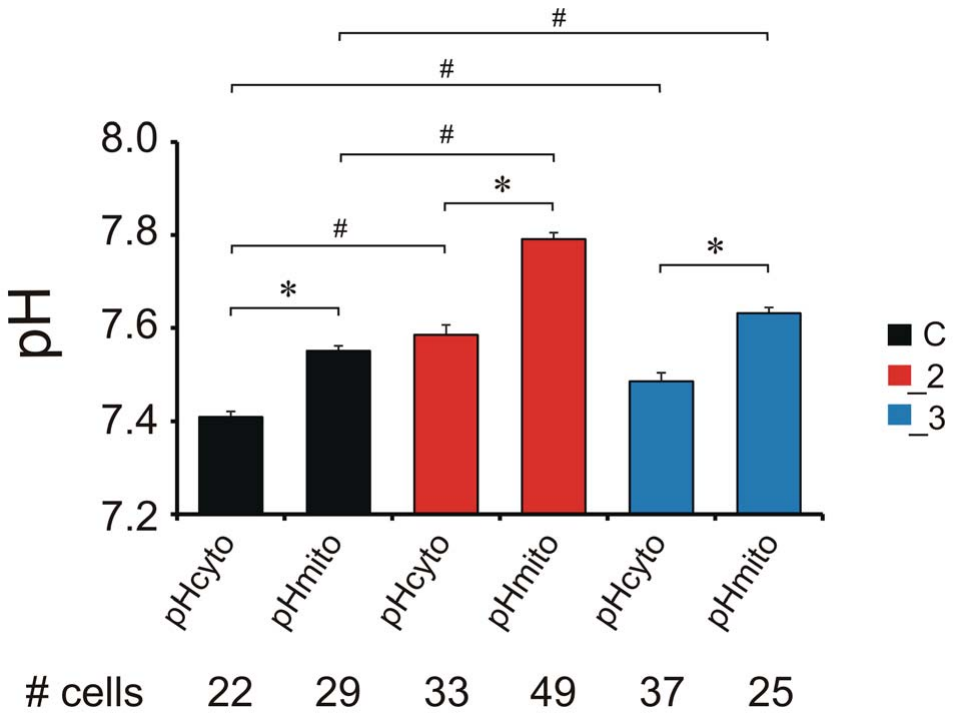
Single cell characterization of cellular pH in steady-state conditions. Average resting pH_mito_ and pH_cyto_ measured by simultaneous imaging of mitoSypHer and SNARF fluorescence, respectively. * P<0.05, pH_mito_ vs. pH_cyto_ within each line; ^#^ P<0.05, PMCA-deficient lines vs. control cells.

First, we tested the properties of mitoSypHer in our modified differentiated PC12 cells: mock transfected control line (C), PMCA2-downregulated line (_2) or PMCA3-downregulated line (_3). When transfected, mitoSypHer was expressed at comparable level in all examined PC12 lines (Fig. S1A) and efficiently targeted to mitochondrial matrix, as demonstrated by enriched reactivity for an anti-GFP antibody and its high colocalization with Mito Tracker Red (Fig. S1B, Pearson coefficients: 0.89±0.07 for C, n = 6; 0.91±0.08 for _2, n = 8; 0.86±0.04 for _3, n = 6). The transfection did not affect viability of the cells, which was in the range of 88–95% (data not shown). The mitoSypHer probe allows for dynamic pH measurement by monitoring the opposite changes in fluorescence at λ_ex_ = 430 and λ_ex_ = 485. To verify if PMCA2 or PMCA3 reduction could affect mitoSypHer spectral properties we performed *in situ* calibration, which showed a Hill slope of 0.95±0.1, 0.92±0.16 and 0.89±0.09 for C, _2 and _3 lines, respectively (Fig. S1C). The switch from pH 7 to 10 resulted in impressive 18-fold increase in 485/430 ratio in all lines whereas in the pH range of 7–8 the observed rise was nearly 4-fold. The calibration curves of mitoSypHer in our differentiated PC12 lines closely matched those obtained in HeLa cells [13], demonstrating that mitoSypHer response was unaltered by PMCAs downregulation. Having validated the probe, we next performed simultaneous measurements of resting pH_mito_ and pH_cyto_ in single cells (Fig. 2A) using mitoSypHer and cytosolic red-shifted fluorescent dye SNARF (5-(and 6)-carboxy-SNARF-1). The spectral properties of these indicators do not overlap allowing for efficient discrimination between pH changes between mitochondria and cytosol. Resting mitochondrial pH in _2 (7.78±0.01) and _3 (7.62±0.01) lines was notably higher in comparison to C (7.53±0.02). Changes in pH_cyto_ were in parallel to pH_mito_ with the highest value noted in _2 (7.56±0.02), followed by _3 (7.49±0.02) and control cells (7.41±0.03). In overall, the cytosolic pH was lower than mitochondrial in each line measured, consistent with chemiosmotic coupling hypothesis and experimental data [13]. As a consequence, pH gradient across the inner mitochondrial membrane (ΔpH = pH_mito_−pH_cyto_) was higher in _2 line.

### PMCA2 and PMCA3 modulate the amplitude of K^+^-evoked pH changes through regulation of intracellular Ca^2+^ load

**Figure 3:**
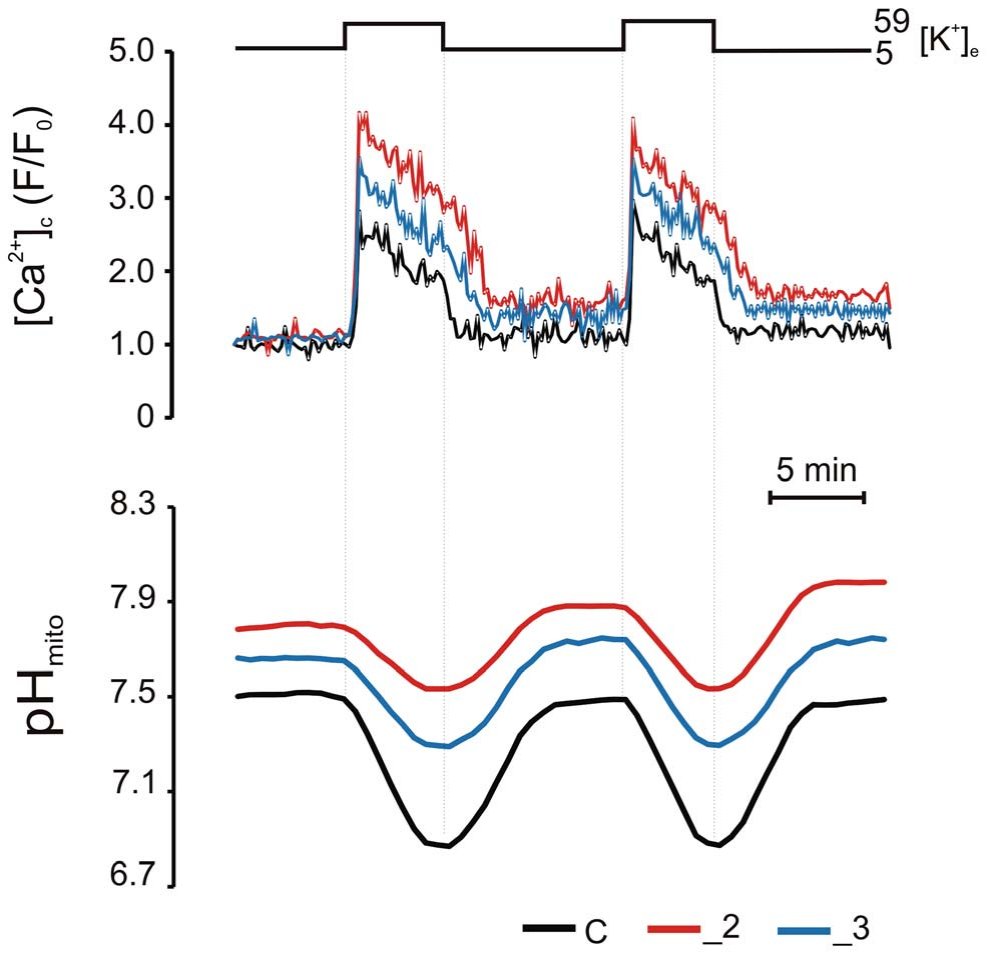
Ca^2+^- and PMCA-dependent mitochondrial acidification. Cells expressing mitoSypHer were loaded with 10 µM Fura-2 for 1 h and simultaneous changes in Fura-2/mitoSypHer fluorescence were recorded in single cells. The traces show the mean response of (Ca^2+^)_c_ (upper panel) and pH_mito_ (lower panel) in n = 11 cells for C, _2, _3 lines following repetitive 59 mM KCl stimulation and recovery.

To evaluate the effects of PMCA2 or PMCA3 reduction on pH changes during KCl-evoked Ca^2+^ loads, PC12 lines expressing mitoSypHer were loaded with Fura-2 for simultaneous recording of (Ca^2+^)_c_ and pH_mito_. Depolarizing concentration of KCL was chosen because (i) it can mimic action potential-driven activation of voltage-dependent Ca^2+^ channels (VDCCs) and Ca^2+^ release from intracellular stores and (ii) PMCA is largely responsible for (Ca^2+^)_i_ restoration after such stimulation [30]. We observed, that cytosolic Ca^2+^ elevations evoked by repetitive treatment with 59 mM KCl were in parallel with mitochondrial acidification (Fig. 3), however the magnitude of pH_mito_ drop was PMCAs-dependent. Interestingly, the degree of acidification in modified lines was inversely correlated with KCl-induced Ca^2+^ load. While PMCA2- or PMCA3-reduction potentiated KCl-evoked (Ca^2+^)_c_ transients by 60±18% and by 32±13% in _2 and _3, respectively, during each stimulation the absolute pH_mito_ response, in relation to control, was reduced by 54±6% in _2 line and by 35±11% in _3 line.

**Figure 4:**
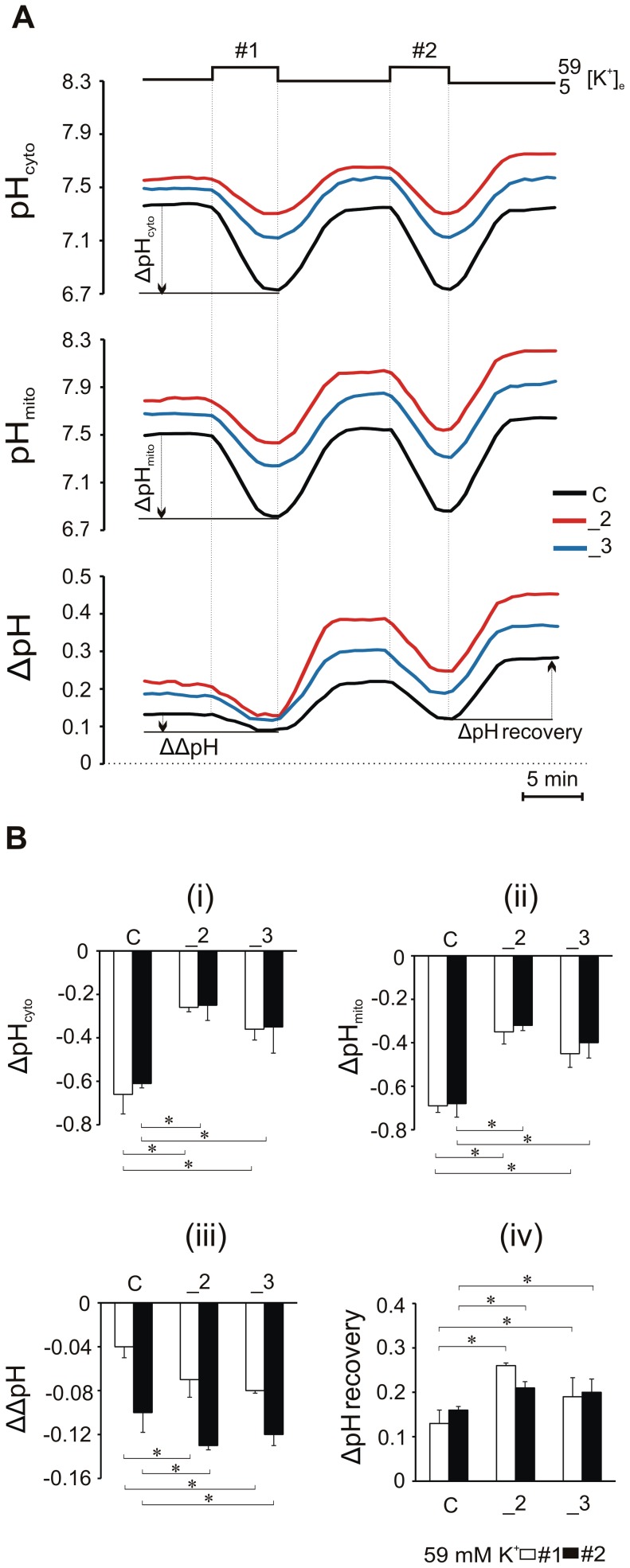
Changes in the mitochondrial pH gradient (ΔpH) during and after 59 mM KCl stimulation. (A) Simultaneous recordings of pH_cyto_ and pH_mito_ in cells expressing mitoSypHer loaded with 10 µM SNARF for 40 min. The cells were imaged in resting conditions (5 mM KCl) and following repetitive stimulation with KCl (59 mM). For each measurement, ΔpH was estimated as pH_mito_−pH_cyto_. (B) average changes in pH_cyto_ (i), pH_mito_ (ii), ΔpH (iii) during each KCl stimulation and in ΔpH after KCl removal (iv) from n = 24, n = 26, n = 23 cells for C, _2, _3 lines respectively. * P<0.05, PMCA-deficient lines vs. control cells.

To follow ΔpH changes during KCl treatment we switched back to the concurrent recordings of pH_mito_ and pH_cyto_ (Fig. 4A). In all lines measured, the monophasic decay in pH_mito_ typically exceeded those in the cytosol. The drop in pH_cyto_ (Fig. 4B, i) and pH_mito_ again (Fig. 4B, ii) was of smaller magnitude in _2 and _3 cells, but during each KCl stimulation ΔpH was undergoing more pronounced reductions in these cells than in control (Fig. 4, iii). The larger decrease in ΔpH was also observed during second stimulation. Upon KCl withdrawal, pH_cyto_ returned to the resting baseline, whereas pH_mito_ typically surpassed its pre-stimulatory level. As a result, ΔpH was 0.16±0.08, 0.22±0.01, 0.21±0.03 pH unit higher than in “quiescent” C, _2 and _3 cells, respectively, following recovery from the second stimulation (Fig. 4, iv).

### PMCA2 and PMCA3 are the main sources of intracellular protons during (Ca^2+^)_c_ elevations generated by Ca^2+^ entry through VDCCs

**Figure 5:**
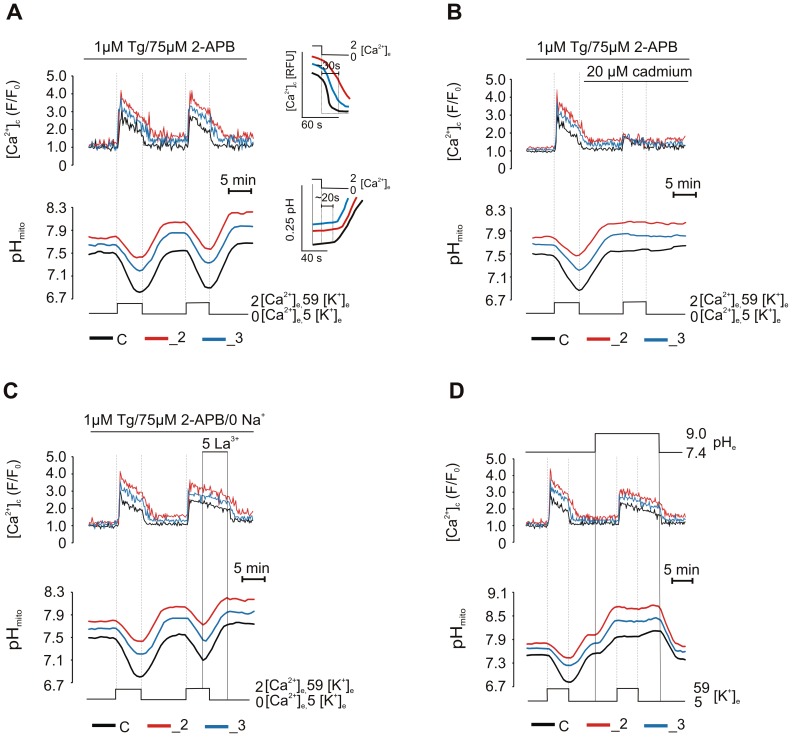
The response of mitochondrial pH to the inhibition of an efflux and the release component of (Ca^2+^)_c_ elevations. (A) The average effect of extracellular Ca^2+^ removal and its subsequent restitution on (Ca^2+^)_c_ and pH_mito_ changes in n = 18, n = 12, n = 21 cells for C, _2, _3 lines respectively, pretreated for 20 min with thapsigargin and 2-APB. The insets show SERCA-independent (Ca^2+^)_c_ clearance (upper) and pH_mito_ recovery (lower). (B) The average effect of cadmium (VDCCs inhibitor) in n = 29, n = 19, n = 22 cells for C, _2, _3 lines respectively, on KCl-evoked (Ca^2+^)_c_ influx and concomitant pH_mito_ changes. (C) The average effect of 5 mM La^3+^ in n = 16 cells for C, _2, _3 lines showing delay in (Ca^2+^)_c_ clearance and pH_mito_ alkalization under low extracellular Na^+^. (D) The average effect of extracellular alkaline pH (9.0) followed by pH return to 7.4 in n = 20, n = 12, n = 20 cells for C, _2, _3 lines respectively, on (Ca^2+^)_c_ elevations and pH_mito_ changes.

**Figure 6:**
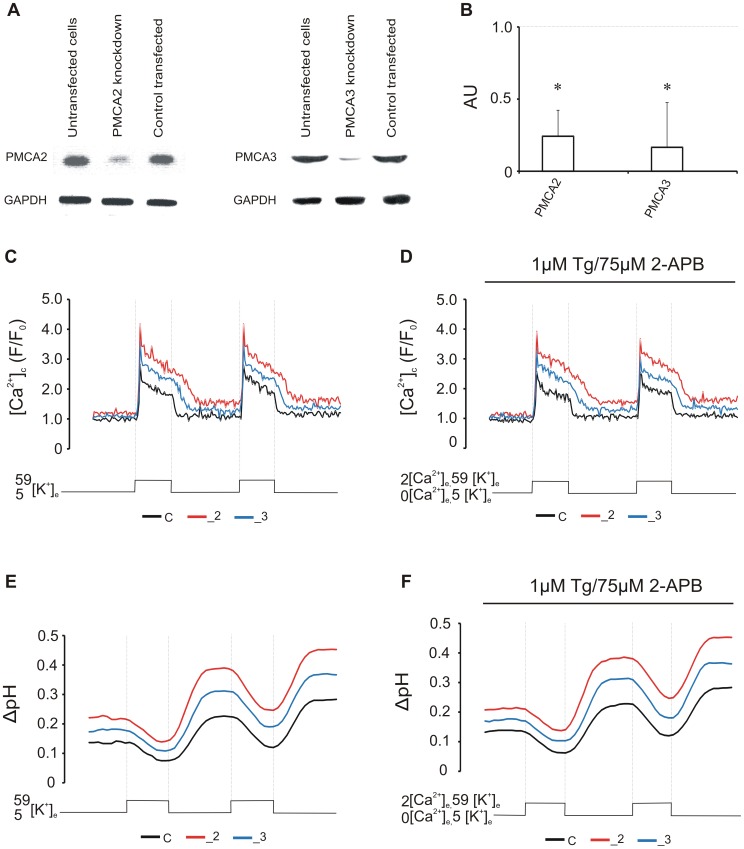
Changes in (Ca^2+^)_c_ and ΔpH are reproduced in transiently transfected cells. (A) Immunodetection of PMCA2 or PMCA3 in PC12 cells transiently transfected with phosphothioate oligodeoxynucleotide probes. (B) Densitometric analysis of PMCAs showing ∼75% knock-down of target genes. The results are presented as arbitrary units (AU) obtained after normalization to endogenous GAPDH level. The dotted line shows the value for untransfected cells (control cells). * P<0.05 PMCA-downregulated cells vs. control cells. (C) The effect of transient PMCAs silencing on (Ca^2+^)_c_ in n = 17 cells for each line without or (D) with the presence of thapsigargin and 2-APB in n = 20 cells for each line. (E) corresponding changes in ΔpH without or (F) with the presence of thapsigargin and 2-APB.

To determine the contribution of particular Ca^2+^ handling systems to cellular acidification, we next treated cells with thapsigargin (Tg) to inhibit sarco(endo)plasmic reticulum Ca^2+^-ATPase (SERCA) and with 2-APB (2-Aminoethoxydiphenyl borate), an inhibitor of store-operated calcium channels and IP_3_ receptor. Under low extracellular Ca^2+^, Tg will deplete the ER and 2-APB will block its repletion through store-operated calcium entry (SOCE), when Ca^2+^ will be restituted. As shown, in Fig. 5A re-addition of external Ca^2+^ together with KCl showed a massive Ca^2+^ influx and concomitant decrease in pH_mito_ with the magnitude comparable to non-inhibitory conditions. Moreover, we observed that PMCAs downregulation slowed down Ca^2+^ clearance following extracellular Ca^2+^ removal, and the pH recovery was delayed until (Ca^2+^)_c_ was nearly at the resting level (Fig. 5A, insets). Thus, SOCE is not required for Ca^2+^-dependent mitochondrial acidification and SERCA is not a main producer of intracellular H^+^ in our experimental model. Additional experiments with transiently transfected cells confirmed that PMCA2 or PMCA3 knockdown affected cellular pH response to KCl-induced (Ca^2+^)_c_ influx and similar profiles of (Ca^2+^)_c_ and ΔpH changes were observed in conditions with or without Tg (Fig. 6). This strengthen our conclusion regarding predominant role of neuro-specific PMCA isoforms in the regulation of pH excursions in PC12 cells.

Because the stimulating effect of KCl results from membrane depolarization with subsequent opening of VDCCs [31], we next examined if the activation of these channels may represent a main source of Ca^2+^ influx. Indeed, blockage of voltage-dependent calcium current by cadmium markedly reduced the amplitude of Ca^2+^ transients and completely abolished subsequent intracellular pH changes (Fig. 5B).

Further, we attempted to verify NCX role by treating cells with Tg and replacing Na^+^ with Li^+^ in a buffer (Fig. 5C). The pH curves during first KCl stimulation matching those obtained under non-inhibitory conditions indicated neglectable NCX participation in observed pH changes. These conditions also allowed us to refine the activity of PMCA, which was directly proportional to the rate of (Ca^2+^)_c_ decrease upon the removal of extracellular Ca^2+^. La^3+^ (5 mM), which is known to inhibit PMCA, added during second stimulation blocked Ca^2+^ clearance and resulted in mitochondrial alkalinization even under low extracellular K^+^. We also inhibited PMCA by reducing the availability of H^+^ to be exchanged with cytosolic Ca^2+^, by increasing extracellular pH up to 9 (Fig. 5D). This markedly delayed Ca^2+^ recovery upon KCl removal, whereas pH restoration to 7.4 resulted in a rapid activation of (Ca^2+^)_c_ clearance, pointing out inhibition of PMCA under high extracellular pH. Also, alkaline pH completely attenuated KCl-induced pH_mito_ decrease in all lines. Because all the conditions that inhibited PMCA every time decreased cellular acidification, PMCA may be considered as a main source of intracellular H^+^ during KCL-evoked Ca^2+^ loads. Thus, markedly decreased Ca^2+^-induced pH response in _2 and _3 lines can be attributed to diminished level of PMCA2 and PMCA3 isoforms.

### Electron transport chain contributes to PMCA-dependent mitochondrial H^+^ fluxes

**Figure 7:**
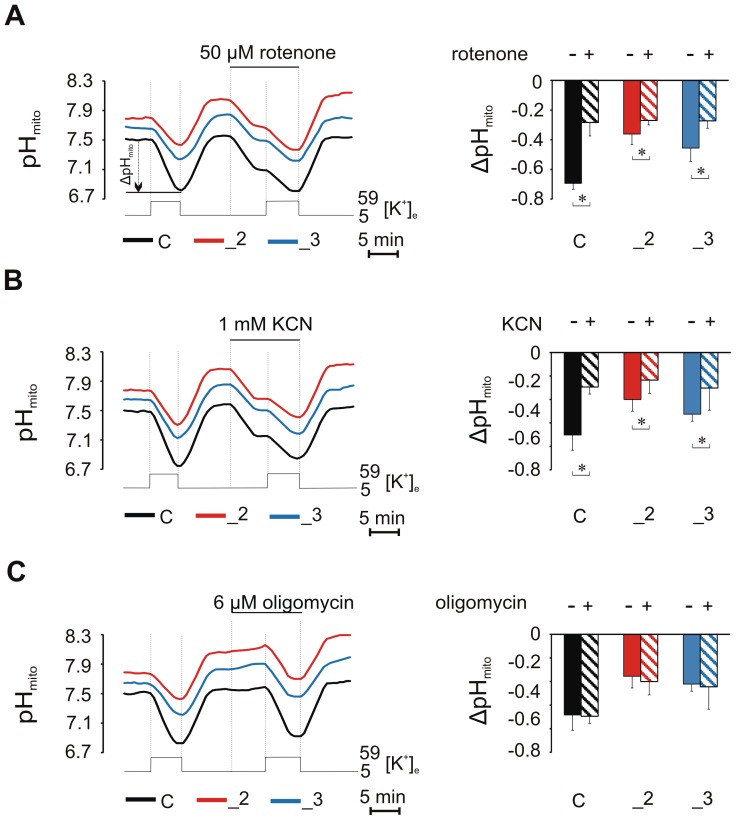
Contribution of electron transport chain (ETC) to mitochondrial H^+^ fluxes. The traces showing the effect of rotenone (A), KCN (B) or oligomycin (C) on 59 mM KCl-evoked pH_mito_ responses in n = 10, n = 13, n = 15 for C, _2, _3 lines, respectively. The column graphs show the drug effects on pH_mito_ loss. * P<0.05 drug treated vs. untreated.

Because PMCAs-dependent acidification of mitochondria during (Ca^2+^)_c_ transients was shown in this study to occur in parallel with cytosolic pH drop, we attempted to evaluate if the electron transport chain (ETC) may regulate cytosolic H^+^ influx to the matrix. We first blocked ETC with rotenone (inhibitor of complex I) or KCN (inhibitor of complex IV). Application of inhibitors alone caused immediate decrease in pH_mito_ in all lines, matching the pH_mito_ response during first KCl stimulation before the inhibitors were added (compare first and second KCl stimulation in Fig. 7 A and B). Additionally, each of the inhibitors reduced KCl-evoked pH_mito_ decrease to 40±9% in C, 74±5% in _2 and to 59±7% in _3 of the value noted in these lines when no inhibitors were present. We then blocked ATP synthase by oligomycin (Fig. 7C). However, we did not observe expected pH_mito_ increase over 5-min incubation period possibly due to maximal alkalization of mitochondria following first KCl stimulation. In each line, oligomycin exerted only moderate effect on the magnitude of Ca^2+^-dependent pH_mito_ decrease.

### Increased ΔpH coincided with elevated TMRE fluorescence in PMCA2 knock-down cells

**Figure 8:**
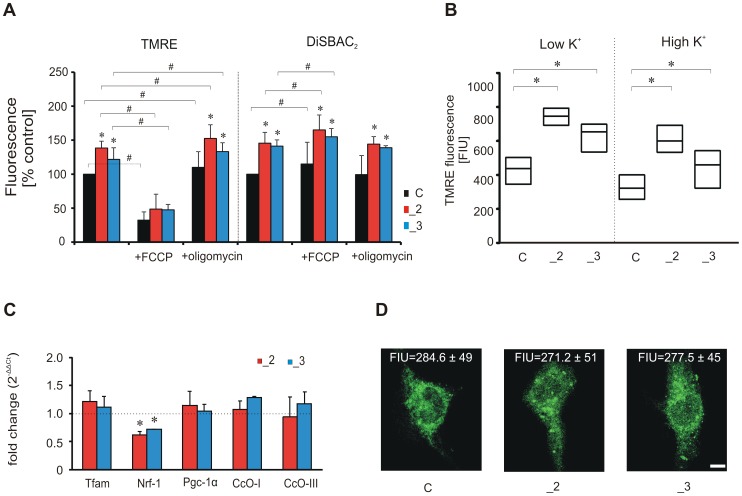
The relative contribution of ΔΨ_m_ and ΔΨ_p_ to TMRE fluorescence. (A) The effect of 10 min preincubation with FCCP (1 µM) or oligomycin (6 µM) on TMRE or DiSBAC_2_ fluorescence assessed by flow cytometry in 10^4^ cells. The fluorescence level in non-treated cells was taken as 100%. * P<0.05, PMCA-deficient lines vs. control cells within inhibitor treated or non-treated groups; ^#^ P<0.05, inhibitor treated cells vs. non-treated. (B) The dependence of increased TMRE on ΔΨ_p_. Before experiment, the medium was exchanged for Ca^2+^-free buffer (20 mM HEPES, pH 7.4, 2 mM CaCl_2_, 150 mM NaCl, 5 mM KCl, 1 mM MgCl_2_, 10 mM glucose) containing either 5 mM (low K^+^) or 59 mM (high K^+^) KCl, in which cells were incubated for 5 min before the addition of TMRE. After 10 min loading period, cellular TMRE fluorescence was acquired. Data are presented in median/quartiles and represent average values from 10^4^ cells. *P<0.05, PMCA-deficient cells vs. control. (C) Real-time PCR analysis of mitochondrial biogenesis markers (Tfam, Nrf-1 and Pgc-1α) and mitochondrially encoded subunits I and III of cytochrome c oxidase (CcO). The expression level of each gene in control line was taken as 1 (dotted line). The relative fold change after normalization to Gapdh expression is shown. * P<0.05, PMCA-deficient lines vs. control cells. (D) Evaluation of mitochondrial mass with MitoTracker Green TM in fixed cells using TCS S5 confocal microscope. The average fluorescence from n = 9, n = 11, n = 14 cells for C, _2, _3 lines, respectively, was measured with microscope accompanying software. Scale bar 10 µm. FIU – fluorescence intensity units.

Based on the results obtained from ΔpH imaging, we next measured mitochondrial membrane potential (ΔΨ_m_), which is thought to reflect mitochondrial energization state. By using nonquenching concentration of TMRE (25 nM) we also determined whether PMCA2- or PMCA3-reduction may trigger depolarization during (Ca^2+^)_c_ transients. First, we observed increased TMRE fluorescence intensity in _2 and _3 cells in steady-state conditions in relation to control (Fig. 8A). Because TMRE uptake is sensitive to changes in either ΔΨ_m_ and plasma membrane potential (ΔΨ_p_), in parallel experiment we monitored ΔΨ_p_ using DiSBAC_2_. Elevated ΔΨ_p_-related fluorescence observed in _2 and _3 lines indicated altered plasma membrane potential (Fig. 8A). To distinguish the relative contribution of ΔΨ_m_ and ΔΨ_p_ to observed TMRE fluorescence increase, we depolarized Ψ_p_ with 59 mM KCl before loading with TMRE (Fig. 8B). Pre-treatment with high K^+^ exerted, however, only a small effect on TMRE suggesting that differences in signal intensity between lines were due to ΔΨ_p_. Additionally, TMRE uptake was not affected by increased mitochondrial biogenesis, as neither changes in expression of Tfam, Nrf-1 and Pgc-1α considered as mitochondrial biogenesis markers nor mitochondrially-encoded subunits I and III of cytochrome c-oxidase reflecting the copy number of mitochondrial DNA were detected (Fig. 8C). In addition, mitochondrial mass was unchanged in PMCA-deficient lines, as evaluated using Mitotracker Green FM probe (Fig. 8D).

**Figure 9:**
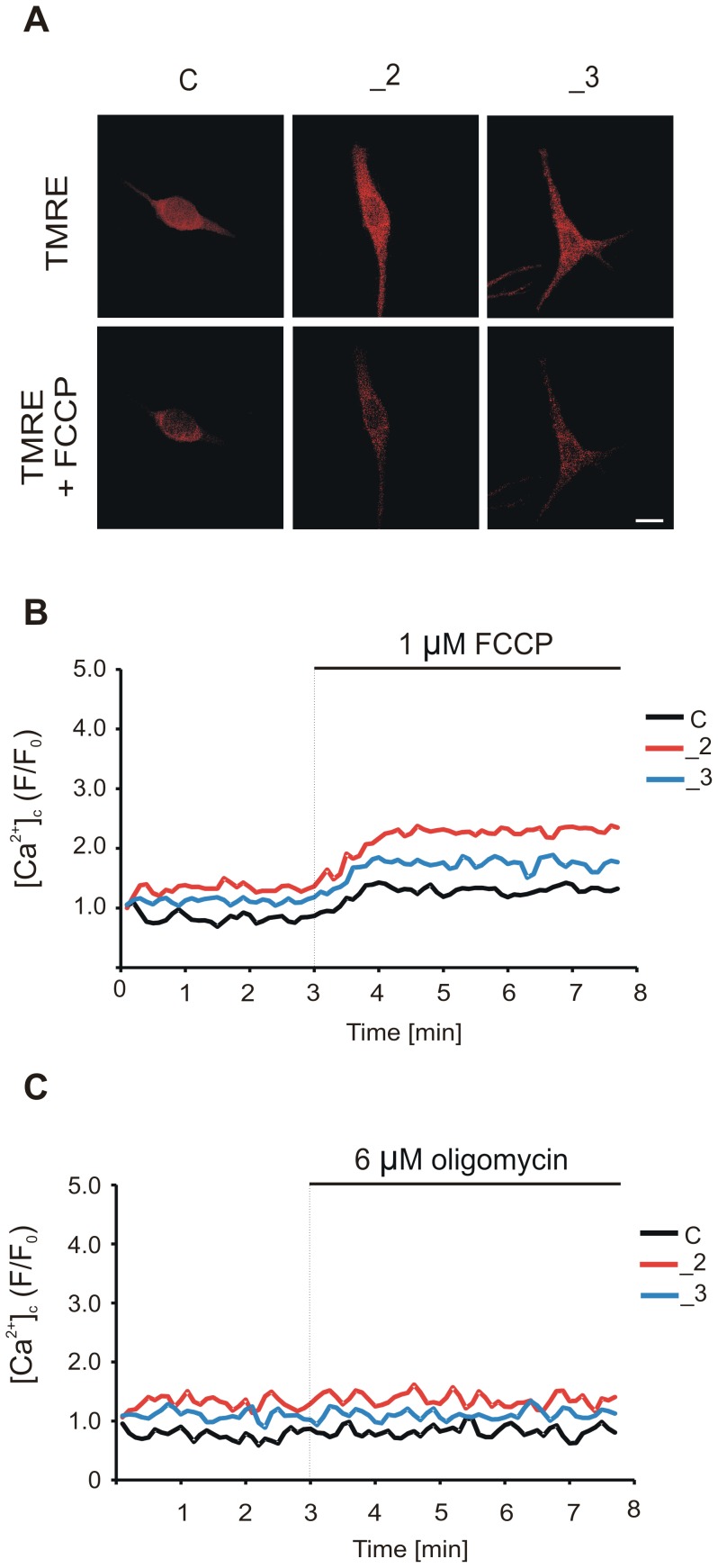
TMRE fluorescence decay upon FCCP treatment and the effect on (Ca^2+^)_c_. (A) The representative micrographs showing a decay in TMRE fluorescence in a single cell after 30 s depolarization with 1 µM FCCP. Scale bar 10 µm. (B) Representative traces showing average FCCP-induced Fura-2 fluorescence increase due to release of mitochondrial Ca^2+^ in n = 15, n = 20, n = 19 cells for C, _2, _3 lines, respectively. (C) Representative traces of average Fura-2 fluorescence showing lack of 6 µM oligomycin effect on (Ca^2+^)_c_ in 14 cells.

To ensure that higher TMRE signal was not a result of dye release during loading and consequent unqenching, TMRE-loaded cells were treated with protonophore FCCP (1 µM) or oligomycin (6 µM). FCCP-induced depolarization resulted in massive decrease in TMRE signal in all lines coinciding with a slight increase in ΔΨ_p_. Application of oligomycin used to block protons re-entry into the matrix caused a small but significant ΔΨ_m_ hyperpolarization notably higher in _2 and _3 lines without affecting ΔΨ_p_. The loss of punctuate TMRE signal, as a result of TMRE release during depolarization by FCCP, was also observed in individual cells (Fig. 9A). Moreover, FCCP evoked a significantly higher rise in (Ca^2+^)_c_ in _2 and _3 lines than in control (Fig. 9B), whereas the application of oligomycin did not change (Ca^2+^)_c_ (Fig. 9C). This demonstrates that basal state of mitochondrial Ca^2+^ loading is increased in PMCA-deficient cells, particularly in _2 line.

### KCl-evoked ΔΨ_m_ depolarizations in PMCA2-deficient line are blocked by cyclosporin A or bongkrekic acid

**Figure 10:**
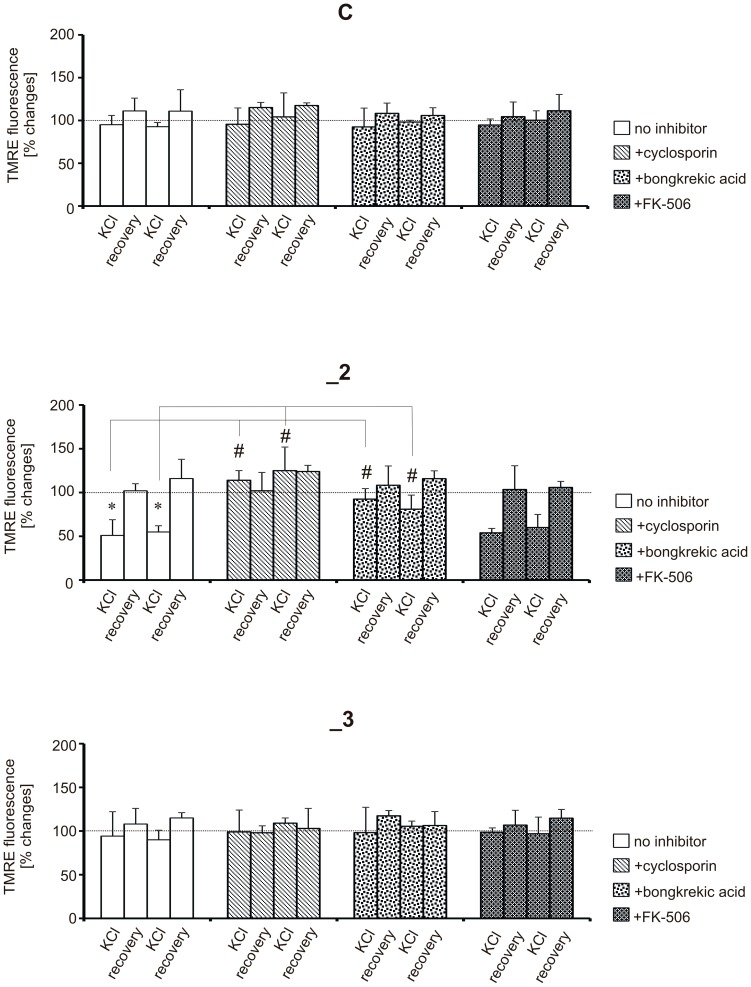
The effect of 59ΔΨ_m_ depolarizations. ΔΨ_m_ changes were measured 10 min after first KCl stimulation, 10 min after first KCl removal (first recovery phase), 10 min after second KCl stimulation and 10 min after second KCl removal (second recovery phase). The level of TMRE fluorescence in resting conditions (5 mM KCl) in each line was taken as 100% (dotted line). Cyclosporin (1 µM), bongkrekic acid (10 µM) or FK-506 (10 µM) were added 1 h before first KCl stimulation. * P<0.05, KCl stimulated vs. resting cells; ^#^ P<0.05, inhibitor treated cells vs. non-treated.

To evaluate whether (Ca^2+^)_c_ elevations can affect ΔΨ_m_, TMRE fluorescence was measured at selected time points of KCl stimulation or recovery, at which ΔpH alterations were the most pronounced: 10 min after 1^st^ KCl stimulation, 10 min after KCl removal (1^st^ recovery phase), 10 min after 2^nd^ KCl stimulation and 10 min after 2^nd^ KCl removal (2^nd^ recovery phase). In control and _3 line, we observed only minor alterations in ΔΨ_m_ during KCl treatment but these little depolarization events did not correlate with the amplitude of (Ca^2+^)_c_ transients, and were considered as insignificant. In contrast, in _2 line each KCl stimulation evoked ΔΨ_m_ depolarization with subsequent decrease in TMRE fluorescence by 51±18% in relation to resting level. (Fig. 10). We then treated cells with cyclosporin A (CsA), a potent inhibitor of mPTP, which fully rescued the reduced TMRE fluorescence. Because CsA is also a well-known inhibitor of calcineurin [32], we used bongkrekic acid (BA) which inhibits mitochondrial ATP/ADP translocase without affecting calcineurin activity. BA partially rescued the reduced TMRE fluorescence while an inhibitor of calcineurin (FK-506) was not able to preserve mitochondria from ΔΨ_m_ loss during KCl-induced Ca^2+^ loads.

To validate the effects of CsA or BA we induced mitochondrial swelling which correlates with decrease in light scattering. We found that addition of 10 µM Ca^2+^ to the mitochondrial suspension induced a small but significant swelling in _2 line (Fig. S2) whereas atractylate added before Ca^2+^ exposure resulted in extensive swelling in all lines. In both cases, swelling was fully prevented by 1 µM CsA or 10 µM BA, added before Ca^2+^. Therefore, our results indicate that Ca^2+^-driven ΔΨ_m_ collapse in PMCA2-deficient line is mediated through CsA-sensitive mechanism.

## Discussion

Despite the proposed predominant role of PMCA in Ca^2+^-dependent regulation of organellar pH, so far no reports have evaluated the contribution of particular PMCA isoforms to mitochondrial proton gradient. Moreover, no studies have been attempted to answer whether altered PMCA expression and concomitant disturbances in Ca^2+^ signaling may affect intracellular pH. The study on *deafwaddler* mouse indicated that the reduction in PMCA2 expression by half may result in motor neuron dysfunctions and mediate neuronal death [33]. Therefore, to avoid dramatic compromise on cellular viability, we have obtained homogenous neuron-like PC12 stably transfected clones with nearly 50% decrease in PMCA2 or PMCA3 protein, with yet no visible symptoms of increased mortality. This allowed us to analyze if neuron-specific PMCA isoforms modulate Ca^2+^-driven intracellular pH changes.

The resting mitochondrial pH in our PC12 lines was lower in comparison to certain cell lines [34]–[36] but similar to the values reported in other [37], [38]. Therefore, it seems that particular cell types maintain different resting pH to fulfill their specific functional requirements. The additional differences in intracellular pH were seen between our PC12 lines: the highest pH_mito_ and pH_cyto_ values were noted in _2 line, followed by _3 and control. Heterogeneous increase in basal pH_mito_ was observed in HeLa cells and primary cultured neurons upon stimulation with Ca^2+^-mobilizing agents [34]. Elevated pH_mito_ and pH_cyto_ demonstrated in our PMCA-downregulated cells in steady-state conditions also suggested a dependence on calcium level. Indeed, our previous study has shown that PMCA2 or PMCA3 reduction caused an increase in resting (Ca^2+^)_c_ [39]. Because PMCA transports large quantities of protons during Ca^2+^ extrusion, parallel acidification of cytosol and mitochondria is expected if the activity of PMCA remains unaffected. Such a phenomenon has been demonstrated in cortical neurons stimulated with glutamate [12]. Based on PMCA/pH relationship, the extent of matrix alkalization in _2 and _3 lines may reflect reduced level (and activity) of neuro-specific isoforms. We suppose that the knock-down of PMCA2 or PMCA3 which are counted as fast reacting, dramatically reduce the amount of H^+^ entering cytosol leading to pH_mito_ increase. The regulation of mitochondrial pH and function by cytosolic Ca^2+^ transients requires the uptake of Ca^2+^ to mitochondria and both Ca^2+^-dependent alkalization or acidification of matrix have been demonstrated [38], [40], [41]. The accumulation of Ca^2+^ depends on ΔΨm-driven electrochemical Ca^2+^ gradient and the gradient of this ion between cytosol and mitochondria. Whether Ca^2+^ uptake into mitochondria is through mitochondrial uniporter or Ca^2+^/H^+^ exchanger, it should depolarize energized mitochondria (reviewed in [6]).

Tetramethylrhodamine probes have been widely used to monitor ΔΨ_m_ [42], [43]. However, TMRE uptake is also sensitive to ΔΨp which may impact the amount of TMRE entering the cytoplasmic space, thereby affecting how much dye is available for mitochondria. Therefore, to resolve the potential contribution of ΔΨ_p_ and ΔΨ_m_ to increased TMRE fluorescence in _2 and _3 lines we depolarized plasma membrane with 59 mM K^+^ before TMRE loading. This strategy was also used by Krohn et. al. [44] or by Perry et. al. [45]. Taking into account the Nernstian behavior of TMRE probe and the results presented in Fig. 8B, one may suggest that even in the presence of high K^+^, elevated TMRE fluorescence is almost entirely dependent on ΔΨ_p_. It is in agreement with ΔΨ_p_ and/or ΔΨ_m_ dependency of TMRE uptake. We confirmed the reliability of TMRE to measure membrane potential by using FCCP and oligomycin. It is known that lower FCCP concentrations will specifically collapse ΔΨ_m_, while high concentrations (>2.5 µM) will also significantly diminish ΔΨ_p_ [43]. However, this effect is likely to be variable with cell type. In our study we applied 1 µM FCCP and observed slight hyperpolarization of ΔΨ_p_, similarly to the effect reported in [46]. The paradoxical FCCP-induced increase in DiSBAC_2_ signal can also be due to the fact that plasma membrane potential is created not by proton pump so protonophore cannot short-circuit it. Instead, FCCP equilibrates pH across plasma membrane carrying positively charged protons from cytoplasm to outside medium thus generating higher membrane potential. Treatment with oligomycin caused a moderate increase in pH_mito_ what is consistent with slight hyperpolarization shown in Fig. 8A. This indicates low rate of ATP turnover and state of mitochondria close to state 4.

In agreement with the prediction that mitochondrial Ca^2+^ uptake may elevate ΔpH if Ca^2+^ charge is compensated by protons moving through ETC, we found ΔpH to be increased in _2 line. The observed raise in matrix pH in steady-state conditions could then result from charge compensation by the respiratory chain. It is attractive to propose that mitochondrial Ca^2+^ accumulation in _2 line will represent a major trigger coupling pH changes to the rate of ATP synthesis. Indeed, three matrix dehydrogenases activated by (Ca^2+^)_m_ increases [47]–[49] provide reducing equivalents to ETC without affecting matrix acidification. This may reflect increase in PMF when (Ca^2+^)_m_ responds to (Ca^2+^)_c_ elevations. Alternatively, elevation in PMF could result from the inhibition of pathways that dissipate H^+^ gradient. It has been reported that mitochondrial Ca^2+^ uptake may inhibit ATP synthase [50] consequently increasing PMF and reducing ATP level. However, our observations with FCCP and oligomycin as well higher ATP content detected in _2 line (yet unpublished) rather exclude ATP synthase inhibition as a mechanism of PMF increase. Therefore, the increases in pH_mito_ may indicate higher capacity of mitochondria to produce ATP.

In our study we detected pronounced cellular acidification associated with (Ca^2+^)_c_ elevations, however with a PMCAs-dependent magnitude. Bearing in mind that PMCA regulates the amount of protons entering cytosol, downregulation of fast responsive PMCA2 or PMCA3 isoforms may explain weaker pH response in _2 and _3, even despite potentiation of Ca^2+^ influx in these lines during KCl stimulation. Different amplitudes of pH_mito_ decreases between our lines could also reflect altered proton buffering capacity. Higher mitochondrial pH in _2 and _3 lines in steady-state conditions might affect ΔpH drop during KCl stimulation, as reduced buffering pH capacity of mitochondria in the alkaline pH was shown to underlie the loss of ΔpH upon treatment with Ca^2+^ mobilizing agents [reviewed in 6]. Following successive stimulations, we observed the overshoot of ΔpH and a new resting (Ca^2+^)_c_ particularly visible in _2 line. This effect is most likely due to over-activation of mitochondrial matrix dehydrogenases by Ca^2+^ transients but also indicate, that PMCAs-downregulated cells irreversibly lose a substantial part of Ca^2+^ clearing potency. This is additionally supported by the observed KCl-evoked higher Ca^2+^ influx in _2 and _3 lines. Our previous study demonstrated increased expression and concomitantly greater contribution of certain VDCCs to Ca^2+^ influx in PMCAs-deficient lines [39]. Because colocalization of these channels and PMCA has been shown in specific types of neurons [51], we assume their functional relationship in the regulation of Ca^2+^ influx in _2 and _3 cells. It is now apparent that mitochondria of some cell types can accumulate large amounts of Ca^2+^ during membrane depolarization events [52], [53]. Facing mitochondria to domains of high (Ca^2+^)_c_ allows direct mitochondrial Ca^2+^ uptake following VDCCs activation and rapid uptake mode of the mitochondrial uniporter in response to extramitochondrial Ca^2+^ bursts. Nonetheless, even under the disturbed Ca^2+^ homeostasis and despite variations in the absolute cellular pH, all cell lines retained the ability to maintain positive matrix vs. cytosol gradient.

Our data show that the main function of protons transport during Ca^2+^ load can be attributed to PMCA2, and to weaker extend also to PMCA3 because: (1) reduction of their level led to lower degree of mitochondrial acidification as less protons entered cytoplasm; (2) the acidification did not require Ca^2+^ release from internal stores but was related to plasma membrane Ca^2+^ influx through VDCCs; (3) all agents used to inhibit PMCA prevented KCl-induced pH drop and markedly delayed Ca^2+^ clearance.

(Ca^2+^)_c_ elevations and subsequent uptake by mitochondria should result in ΔΨ_m_ dissipation to restrict the availability of mitochondria to synthesize ATP. Decreases in ΔΨ_m_ have been observed in isolated mitochondria exposed to Ca^2+^ overload [54]. In intact cells transient depolarizations have been reported only in some cell types [55]–[57], but not in other [58], [59]. The present study also found no detectable alterations in ΔΨ_m_ in control and _3 lines, despite large pH_mito_ and pH_cyto_ drop during KCl-evoked (Ca^2+^)_c_ elevations. In agreement with the statement that mitochondrial Ca^2+^ uptake must affect ΔΨ_m_, the depolarization events could be too faint to be detected in control and _3 lines. In turn, ΔΨ_m_ depolarizations did occur in _2 cells in response to (Ca^2+^)_c_ elevations. We can assume it could be due to higher ΔΨ_m_ – dependent mitochondrial Ca^2+^ uptake shown in this line, as ΔΨ_m_ – driven elevation of mitochondrial Ca^2+^ may itself dissipate ΔΨ_m_ [60], [61]. Ca^2+^ influx through VDCCs resulting in ΔΨ_m_ loss was shown in CA1 pyramidal cells in hippocampal slides [62]. It also seems possible that a rise in (Ca^2+^)_c_ and then in (Ca^2+^)_m_ may depolarize ΔΨ_m_ through promotion of Ca^2+^ cycling or by decreasing the ATP/ADP·P_i_ ratio due to higher ATP consumption by Ca^2+^-dependent ATPases. This would in turn increase proton backflow to the mitochondrial matrix, depolarizing ΔΨ_m_ and stimulating respiration. The net Ca^2+^ accumulation may occur through the mitochondrial uniporter which activity in neural tissue is particularly high [63]. The entry of positively charged ions could then lower ΔΨ_m_ allowing net H^+^ extrusion by the ETC with the consequent increase in ΔpH. Another possible mechanism may involve Ca^2+^-dependent inhibition of ETC, as was demonstrated in mitochondria exposed to increasing Ca^2+^ concentration [64]–[66]. However, at this stage we are unable to distinguish which portion of ΔΨ_m_ changes during stimulation were due to collapsing of proton gradient or the exchange of charged molecules (e.g. Ca^2+^, P_i_, ADP).

Here, we report that ΔΨ_m_ depolarization in PMCA2-deficient cells is mediated by the activation of CsA-sensitive mechanism. Studies from neuronal and non-neuronal cells suggest that during ion imbalance mitochondria depolarize, swell and release cytochrome c through CsA-sensitive Ca^2+^-activated mPTP opening [67]–[70]. In our model, mitochondrial Ca^2+^ overload may lead to transient mPTP opening resulting in ΔΨ_m_ collapse, outward Ca^2+^ redistribution and matrix acidification. However, contrary to catastrophic nature of mPTP opening, our data demonstrate that ΔΨ_m_ recovered upon KCl removal. This suggests that respiratory chain rebuilt the proton gradient and restored ΔΨ_m_, which may drive Ca^2+^ re-uptake and its gradual accumulation in the matrix. Perhaps, only brief mPTP opening could be sufficient to trigger subsequent death in PMCA2-deficient cells. Because some studies have reported that neuronal mPTP is relatively CsA-insensitive [54], [71], alternative mechanisms such as reactive oxygen species release or adenine nucleotide depletion should also be considered. Additionally, reduced mitochondrial H^+^ concentration may by itself trigger mPTP opening, as an acidic pH was reported to block the opening of mPTP [72], [73]. In line with it, more pronounced acidification observed in control and _3 cells may explain why ΔΨ_m_ is not dissipated in these lines during KCl stimulation.

In summary, we showed that PMCA2 and PMCA3 are responsible for dynamic regulation of cellular pH. In steady-state conditions, concomitant elevation of (Ca^2+^)_c_ and higher Ψm-dependent accumulation of mitochondrial Ca^2+^, and/or decreased influx of cytosolic H^+^ due to PMACA knock-down, may lead to mitochondrial alkalization. It is believable as the amount of H^+^ entering cytosol in exchange for Ca^2+^ seems to depend on the kinetic properties of PMCA isoforms. This could explain why pH response observed during (Ca^2+^)_c_ elevations was modulated in a manner dependent on isoform activity: the smallest response when PMCA2 was downregulated, which is regarded as the fastest reacting, followed by PMCA3 which is only slightly slower than PMCA2. However, during massive Ca^2+^ loads, the potentiation in Ca^2+^ influx observed in _2 line and, as a consequence, mitochondrial Ca^2+^ overload may lead to ΔΨm depolarization. Our data indicate that ΔΨm collapse was triggered by CsA-sensitive mechanism suggesting the involvement of mPTP opening as a possible underlying mechanism. Lack of signs for mPTP formation in _3 cells could indicate that the threshold Ca^2+^ concentration required for Ca^2+^-dependent mPTP opening has not been achieved although an increased Ca^2+^ influx during membrane depolarization was also observed in these cells. The overall data indicate that the relationship between mitochondria and PMCA is much more complex and intimate and exceeds far beyond a simple energetic connection. Our findings provide the evidence, that PMCA membrane composition might be of great importance for preservation of bioenergetic function of mitochondria. Therefore, changes in PMCA expression occurring i.e. in ageing brain or spinal cord injury [74], [75] may profoundly affect cellular metabolic network and disturb mitochondrial function. In view of this, pathological alterations in PMCA expression, in particular PMCA2, may contribute to neurotransmission dysfunctions via a mechanism of mitochondrial depolarization. Undoubtedly, elucidating of the functional interplay between mitochondrial metabolism and neuronal function is of paramount importance for understanding of pathophysiology in various neurological diseases.

## Supporting Information

Figure S1 ***In vitro*** **characterization of mitoSypHer probe in differentiating PC12 cells.** (A) The expression of mitoSypHer vector (i, SypHer) and the corresponding protein content (ii, anti-GFP) assessed using PCR or monoclonal anti-GFP antibodies, respectively. GAPDH was used as an internal control. (B) Confocal images of mitoSypHer (green) in fixed cells labeled with MitoTracker Red (red) showing mitochondrial localization of mitoSypHer (merged). Insets show clear mitochondrial targeting of both probes. Scale bar 20 µm. (C) *In situ* calibration of mitoSypHer obtained by measuring changes in 485/430 ratio with increasing extracellular pH.
(TIF)

Figure S2 **The induction of mitochondrial swelling in the presence of Ca^2+^.** Mitochondrial swelling induced by the addition of 10 µM CaCl_2_ was enhanced by atractylate (20 µM) but inhibited by bongkrekic acid (10 µM) or cyclosporin (1 µM). Swelling was assessed by light absorbance at 520 nm in a suspension of mitochondria. The absorbance at time 0 (before Ca^2+^ exposure) was taken as 100%.
(TIF)
