# Supplementary figures and images for: Plasma Membrane Ca2+-ATPase Isoforms Composition Regulates Cellular pH Homeostasis in Differentiating PC12 Cells in a Manner Dependent on Cytosolic Ca2+ Elevations
Source: PLoS One. 2014 Jul 11;9(7):e102352. doi: 10.1371/journal.pone.0102352 (PMC4094512; doi:10.1371/journal.pone.0102352)

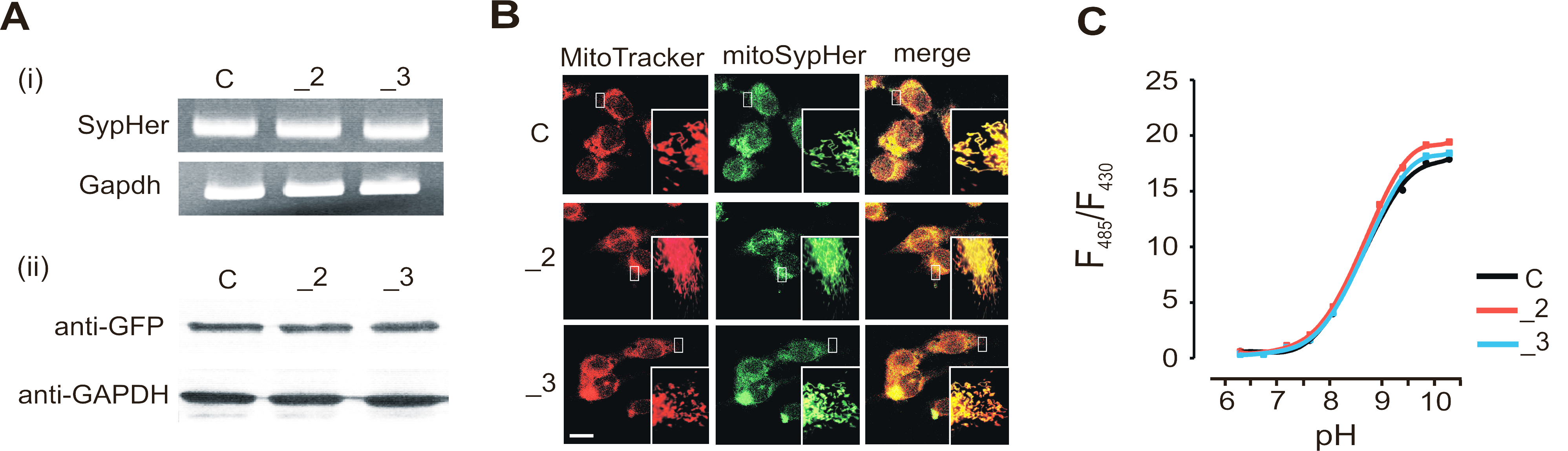

Supplement: Figure S1 — In vitro characterization of mitoSypHer probe in differentiating PC12 cells. (A) The expression of mitoSypHer vector (i, SypHer) and the corresponding protein content (ii, anti-GFP) assessed using PCR or monoclonal anti-GFP antibodies, respectively. GAPDH was used as an internal control. (B) Confocal images of mitoSypHer (green) in fixed cells labeled with MitoTracker Red (red) showing mitochondrial localization of mitoSypHer (merged). Insets show clear mitochondrial targeting of both probes. Scale bar 20 µm. (C) In situ calibration of mitoSypHer obtained by measuring changes in 485/430 ratio with increasing extracellular pH. (TIF) [file pone.0102352.s001.tif]

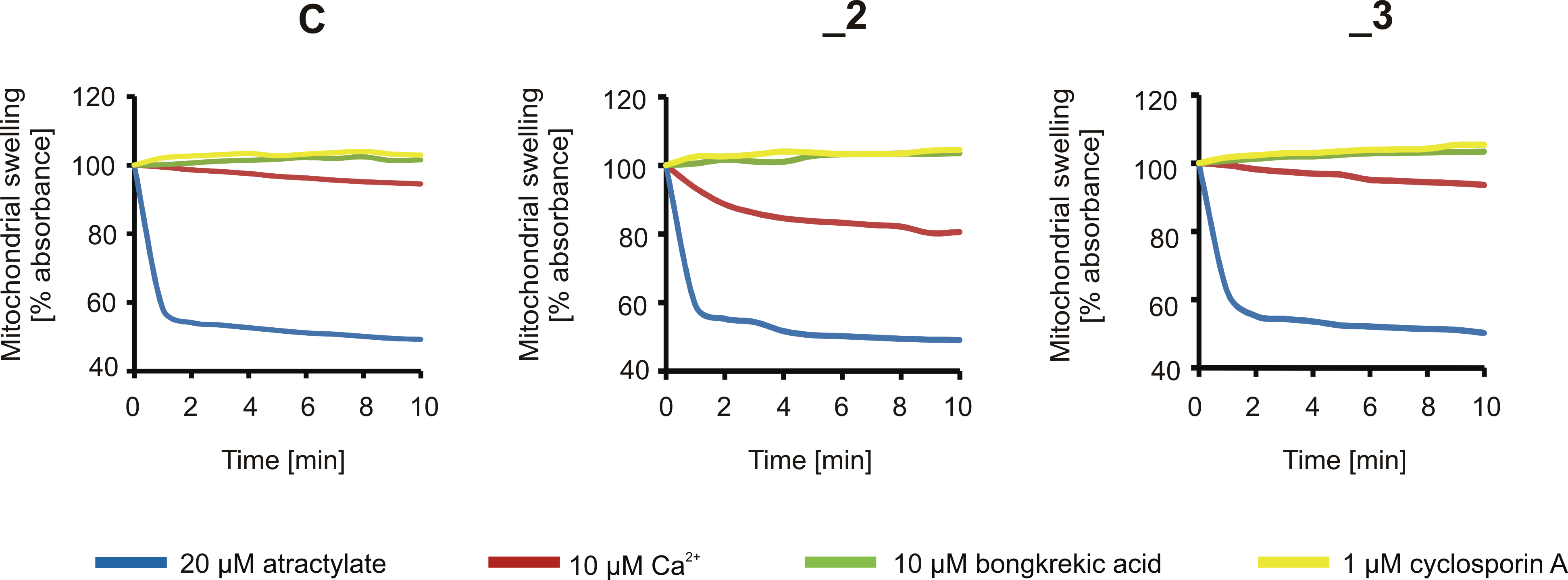

Supplement: Figure S2 — The induction of mitochondrial swelling in the presence of Ca2+. Mitochondrial swelling induced by the addition of 10 µM CaCl2 was enhanced by atractylate (20 µM) but inhibited by bongkrekic acid (10 µM) or cyclosporin (1 µM). Swelling was assessed by light absorbance at 520 nm in a suspension of mitochondria. The absorbance at time 0 (before Ca2+ exposure) was taken as 100%. (TIF) [file pone.0102352.s002.tif]
